# Impact of skeletal muscle loss and sarcopenia on outcomes of neoadjuvant immunochemotherapy in esophageal squamous cell carcinoma

**DOI:** 10.3389/fnut.2025.1650337

**Published:** 2025-09-12

**Authors:** Binwen Xu, Junhong Liu, Yue Zhang, Tao Luo, Jie Xiong, Hanxiao Wang, Guidong Shi, Maoyong Fu

**Affiliations:** Department of Thoracic Surgery, Affiliated Hospital of North Sichuan Medical College, Nanchong, Sichuan, China

**Keywords:** sarcopenia, skeletal muscle index, esophageal squamous cell carcinoma, neoadjuvant immunochemotherapy, overall survival

## Abstract

**Background:**

Sarcopenia is a systemic disorder characterized by the progressive loss of skeletal muscle mass and function; however, its impact on the treatment outcomes of patients with esophageal cancer remains inconclusive. We aimed to evaluate the impact of sarcopenia and dynamic changes in skeletal muscle during treatment on neoadjuvant immunochemotherapy (NICT) efficacy and prognosis in patients with locally advanced ESCC.

**Methods:**

We retrospectively included 272 patients with locally advanced ESCC who received NICT. We calculated the skeletal muscle index (SMI) and its rate of change (ΔSMI%) from CT images at the L3 vertebral level obtained before and after treatment. Sarcopenia was defined as an SMI < 52.4 cm^2^/m^2^ in men and <38.5 cm^2^/m^2^ in women, and a ΔSMI% < −2.8% was designated as excessive skeletal muscle loss.

**Results:**

The prevalence of sarcopenia increased from 50.9% before treatment to 55.1% at therapy completion. Pre-NICT sarcopenia correlated with tumor progression (*p* = 0.02) and was associated with a significantly lower pathological complete response (pCR) in patients who had sarcopenia than in those without (14.7% vs. 25.0%, *p* = 0.04). Patients with tumor progression had a significantly lower SMI than those in the disease-control group (41.6 ± 7.24 vs. 48.71 ± 8.39, *p* = 0.04). In a subgroup analysis of excessive skeletal muscle loss, these patients experienced higher hematologic toxicity (leukopenia: 33.4% vs. 20.9%, *p* = 0.04; anemia: 70.7% vs. 50.6%, *p* = 0.01) and lower pCR rate (12.0% vs. 22.8%, *p* = 0.05). After a median follow-up of 20.4 months, sarcopenia before or after NICT did not significantly affect overall survival (OS) or disease-free survival (DFS) (*p* > 0.05). Conversely, excessive skeletal muscle loss during treatment emerged as an independent prognostic factor for OS in multivariate analysis (HR = 0.47; 95% CI, 0.25–0.91; *p* = 0.03); however, it was not associated with DFS (*p* = 0.22).

**Conclusion:**

Treatment-induced excessive skeletal muscle loss may serve as a predictive marker for NICT toxicity and short-term survival in patients with locally advanced ESCC, highlighting the need for dynamic nutritional monitoring to optimize treatment tolerance.

## Introduction

Esophageal squamous cell carcinoma (ESCC) is a malignancy that poses a significant threat to human health (1). The advent of immune checkpoint inhibitors (ICIs) has made neoadjuvant immunochemotherapy (NICT) an important treatment strategy for patients with locally advanced ESCC (2–4). However, a considerable number of patients demonstrate primary resistance to ICIs and may even experience disease progression (2, 5). Currently, programmed death-ligand 1 (PD-L1) remains the only Food and Drug Administration-approved predictive biomarker for immunotherapy; however, concerns exist regarding its high testing costs and variable accuracy (6). Multiple studies have shown that even PD-L1–negative patients benefit from immunotherapy (2, 7, 8). Therefore, identifying novel predictive markers that are more cost-effective and reliable is urgently required.

Sarcopenia is a systemic disorder characterized by the progressive loss of skeletal muscle mass and function and is closely associated with malnutrition, reduced physical activity, and chronic inflammation (9–11). This condition is recognized as an independent risk factor for poor prognosis across various malignancies (12–14). In patients with ESCC presenting primarily with dysphagia, the reported incidence of sarcopenia ranges from 44.0–74.2%; nonetheless, its treatment-related risks remain frequently underestimated (9, 15–17). Notably, esophageal cancer patients face a risk of nutritional decline from the time of diagnosis, as tumor-mediated metabolic competition, swallowing impairment, and treatment toxicity collectively accelerate muscle loss (18). The impact of sarcopenia on the treatment outcomes of patients with esophageal cancer has been investigated in several studies; however, their findings remain inconclusive. In some studies, it was suggested that sarcopenia is associated with poor prognosis (12–14), while in others, conflicting findings were reported (18–20). Moreover, skeletal muscle mass represents a continuously changing, dynamic parameter. Nevertheless, most current studies rely on cross-sectional assessments at a single time point, and the potential value of dynamic changes in muscle mass during treatment is overlooked. Furthermore, existing evidence primarily pertains to neoadjuvant chemoradiotherapy, with limited studies on immunotherapy.

In this study, we evaluated the effects of sarcopenia and dynamic changes in skeletal muscle mass during treatment on therapeutic response and survival outcomes in patients with locally advanced ESCC undergoing NICT. We further identified independent risk factors for sarcopenia, anticipating that these findings will provide valuable insights for risk stratification and individualized treatment planning for patients with ESCC.

## Materials and methods

### Patients

**Figure 1:**
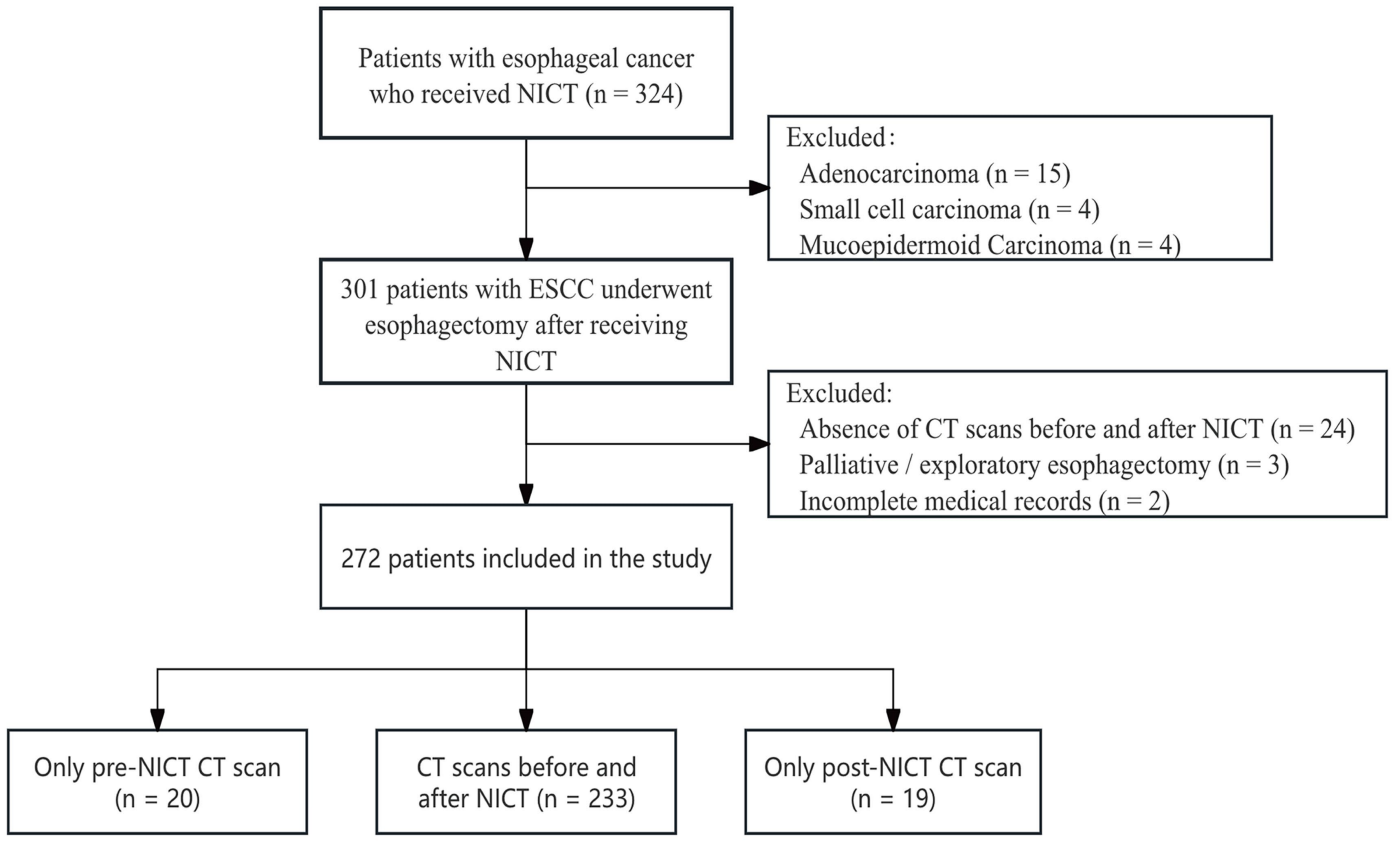
Flow chart of the study.

We retrospectively analyzed the clinical records of patients with esophageal cancer who received neoadjuvant therapy followed by surgical resection at the Affiliated Hospital of North Sichuan Medical College between 2020 and 2024. The institutional ethics committee approved the study (File Number: 2025ER240-1), which was conducted in accordance with the 2013 Declaration of Helsinki; written informed consent was waived owing to its retrospective design. Inclusion criteria were: (i) age 18–80 years and a preoperative diagnosis of locally advanced, resectable ESCC staged at least cT3 or N+; (ii) receipt of at least two cycles of NICT at our institution, followed by minimally invasive McKeown esophagectomy. Exclusion criteria included: (i) prior antitumor treatments or distant metastases; (ii) palliative resection or exploratory surgery only; (iii) lack of abdominal CT images before or after treatment, or incomplete clinical records. Pathological staging was determined following the 8th edition of the Union for International Cancer Control/American Joint Committee on Cancer staging system. Figure 1 shows the patient selection process.

### Assessment of skeletal muscle loss and definition of sarcopenia

We quantified changes in patients’ skeletal muscle mass, using the Skeletal Muscle Index (SMI) and semi-automatically delineated regions of interest (ROIs) in muscle tissue, defined by Hounsfield unit thresholds of −29 to +150 HU, using the SliceOmatic software. We calculated SMI as the total cross-sectional area of all skeletal muscles at the L3 level on pre- and post-treatment abdominal CT scans (cm^2^) divided by height squared (m^2^). Using large-scale population data (21), we defined sarcopenia as an SMI < 52.4 cm^2^/m^2^ in men and <38.5 cm^2^/m^2^ in women. Changes in SMI before and after NICT were expressed as a percentage: ΔSMI% = (SMI_post-NICT – SMI_pre-NICT)/SMI_pre-NICT × 100%. Supplementary Figure 1 presents a single patient’s CT images showing normal muscle mass pre-NICT and sarcopenia post-NICT.

### Neoadjuvant therapy regimens and surgery

The NICT regimen comprised a platinum agent [80 mg/m^2^ intravenously (IV) on day 1] combined with albumin-bound paclitaxel (200 mg/m^2^ IV on day 2), followed by an ICI (200 mg IV on day 3). All patients underwent at least two treatment cycles, with an inter-cycle interval of more than 3 weeks. Upon completing NICT, a multidisciplinary team assessed tumor resectability. For patients deemed suitable for curative resection, a minimally invasive three-incision approach (right thoracic, upper abdominal, and left cervical) was employed, together with a 2.5-field lymphadenectomy (22). Postoperatively, we scheduled follow-up visits every 3 months for the first 2 years and every 6 months thereafter. Nutritional interventions were not standardized in this study; in routine practice, patients with dysphagia underwent dietitian assessment and, if indicated, received oral nutritional supplements or nasogastric tube feeding.

### Endpoints

The primary endpoints were overall survival (OS) and disease-free survival (DFS). We used a Cox proportional hazards regression model to adjust for clinical covariates and evaluate their prognostic significance. OS was defined as the interval between surgery and death from any cause, while DFS was defined as the interval between surgery and first recurrence or death from any cause. Secondary endpoints comprised neoadjuvant treatment-related adverse events, treatment response rate, pathological complete response (pCR), and postoperative complication rate. We employed a multivariate logistic regression model to identify independent risk factors for sarcopenia before and after neoadjuvant therapy. Post-treatment responses were classified following the Response Evaluation Criteria in Solid Tumors (RECIST) as complete response (CR), partial response (PR), stable disease (SD), or progressive disease (PD); CR, PR, and SD were collectively defined as disease control (DC). Treatment-related adverse events (TRAEs) were graded following the guidelines in version 5.0 of the National Cancer Institute’s Common Terminology Criteria for Adverse Events.

### Statistical analysis

Continuous variables that are normally distributed are expressed as mean ± standard deviation (SD). Non-normally distributed variables are expressed as median (interquartile range [IQR]). We compared continuous variables between groups using the Student’s *t*-test or the Wilcoxon rank-sum test, depending on their distribution. The chi-square test or Fisher’s exact test was used to compare categorical variables. OS and DFS were estimated using the Kaplan–Meier method; between-group differences were assessed with the log-rank test, and survival curves were plotted in R version 4.3.2. The Kolmogorov–Smirnov (K–S) test indicated that ΔSMI% followed a normal distribution (*p* < 0.001). We used the Maxstat package in R to determine the optimal ΔSMI% cutoff for OS discrimination. A threshold of −2.8% was identified (*p* < 0.05), and ΔSMI% < −2.8% was defined as excessive skeletal muscle loss. Statistical analyses were performed using SPSS version 25.0 and R version 4.3.2. Two-sided *p*-values < 0.05 were considered statistically significant.

## Results

### Baseline characteristics

We included 272 patients with ESCC treated with NICT followed by surgical resection, with imaging data available for 253 patients before neoadjuvant therapy and for 252 patients after treatment. The baseline clinical characteristics of all patients are shown in Supplementary Table 1. The median age of the patients was 65 years, and the median BMI was 22.9 (IQR 20.8–24.9); most patients were male (73.2%) and reported a history of smoking (52.9%) or alcohol consumption (46.7%). Tumors were located predominantly in the middle third of the esophagus (71.3%) and in the lower third (16.5%). Prior to NICT, clinical staging was predominantly stage III (44.5%), followed by stage II (36.8%).

### Characteristics of sarcopenia and multivariate analysis

Table 1 shows the clinicopathological characteristics of patients with sarcopenia versus those without sarcopenia before and after neoadjuvant therapy. Prior to NICT, 129 patients (50.9%) had sarcopenia, which increased to 139 patients (55.1%) at treatment completion. Supplementary Figure 2 shows that, according to RECIST criteria, those without sarcopenia did not experience PD before or after treatment, and those achieving DC had significantly higher SMI values than did the patients with PD (pre-treatment: 48.71 ± 8.39 vs. 41.60 ± 7.24, *p* = 0.04; post-treatment: 48.43 ± 8.71 vs. 39.57 ± 9.36, *p* = 0.02). Additionally, the pCR rate was higher in patients without sarcopenia before treatment (25.0% vs. 14.7%; *p* = 0.04), although the difference was not significant after treatment (*p* = 0.081 and *p* = 0.197).

**Table 1:** Clinical characteristics.

Variables	Total (*n* = 253)	Pre-NICT sarcopenia		*p*-value	Total (*n* = 252)	Post-NICT sarcopenia		*p*-value
		No (*n* = 124)	Yes (*n* = 129)			No (*n* = 113)	Yes (*n* = 139)	
Age, median, years [IQR]	67(59–70)	66(58–69)	67(60–72)	0.048	67(60–71)	66(58–69)	68(61–72)	0.023
BMI, median, [IQR]	22.9(20.8–24.9)	24.2(22.5–25.9)	21.5(19.9–23.5)	<0.001	22.7(20.7–24.7)	24.1(22.4–25.8)	21.4(19.9–23.5)	<0.001
Sex								
Male	188(74.3%)	79(63.7%)	109(84.5%)	<0.001	188(74.6%)	76(67.3%)	112(80.6%)	0.016
Female	65(25.7%)	45(36.3%)	20(15.5%)		64(25.4%)	37(32.7%)	27(19.4%)	
Smoking								
No	119(47%)	73(58.9%)	46(35.7%)	<0.001	115(45.6%)	57(50.4%)	58(41.7%)	0.167
Yes	134(53%)	51(41.1%)	83(64.3%)		137(54.4%)	56(49.6%)	81(58.3%)	
Drinking								
No	133(52.6%)	79(63.7%)	54(41.9%)	<0.001	131(52.0%)	65(57.5%)	66(47.5%)	0.113
Yes	120(47.4%)	45(36.3%)	75(58.1%)		121(48.0%)	48(42.5%)	73(52.5%)	
Hypertension								
No	201(79.4%)	94(75.8%)	107(82.9%)	0.160	199(79.0%)	84(74.3%)	115(82.7%)	0.104
Yes	52(20.6%)	30(24.2%)	22(17.1%)		53(21.0%)	29(25.7%)	24(17.3%)	
Diabetes								
No	237(93.7%)	113(91.1%)	124(96.1%)	0.103	236(93.7%)	105(92.9%)	131(94.2%)	0.668
Yes	16(6.3%)	11(8.9%)	5(3.9%)		16(6.3%)	8(7.1%)	8(5.8%)	
Cardiopathy								
No	239(94.5%)	116(93.5%)	123(95.3%)	0.531	238(94.4%)	105(92.9%)	133(95.7%)	0.341
Yes	14(5.5%)	8(6.5%)	6(4.7%)		14(5.6%)	8(7.1%)	6(4.3%)	
COPD								
No	226(89.3%)	112(90.3%)	114(88.4%)	0.615	224(88.9%)	100(88.5%)	124(89.2%)	0.858
Yes	27(10.7%)	12(9.7%)	15(11.6%)		28(11.1%)	13(11.5%)	15(10.8%)	
Tumor location								
Upper	32(12.6%)	20(16.1%)	12(9.3%)	0.087	30(11.9%)	12(10.6%)	18(12.9%)	0.721
Middle	180(71.1%)	89(71.8%)	91(70.5%)		181(71.8%)	84(74.3%)	97(69.8%)	
Lower	41(16.2%)	15(12.1%)	26(20.2%)		41(16.3%)	17(15%)	24(17.3%)	
Clinical TNM stage								
II	93(36.8%)	50(40.3%)	43(33.3%)	0.472	183(72.6%)	87(77.0%)	96(69.1%)	0.353
III	112(44.3%)	53(42.7%)	59(45.7%)		57(22.6%)	22(19.5%)	35(31.4%)	
IVA	48(19.0%)	21(16.9%)	27(20.9%)		12(4.8%)	4(3.5%)	8(5.8%)	
Clinical response								
CR + PR	159(62.8%)	76(61.3%)	83(64.3%)	0.026*	162(64.3%)	75(66.4%)	87(62.6%)	0.081*
SD	88(34.8%)	48(38.7%)	40(31.0%)		84(33.3%)	38(33.6%)	46(33.1%)	
PD	6(2.4%)	0	6(4.7%)		6(2.4%)	0	6(2.4%)	
No. of LNs harvested, median, [IQR]	19(13–26)	19(13–26)	20(15–26)	0.660	19(13–26)	18(13–25)	21(13–27)	0.297
Pathological CR								
pCR	50(19.8%)	31(25.0%)	19(14.7%)	0.040	49(19.4%)	26(23.0%)	23(16.5%)	0.197
Non-pCR	203(80.2%)	93(75.0%)	110(85.3%)		203(80.6%)	87(77.0%)	116(83.5%)	
ypTNM stage								
I	108(42.7%)	54(53.5%)	54(41.9%)	0.996	107(42.5%)	49(43.4%)	58(41.7%)	0.859
II	36(14.2%)	17(13.7%)	19(14.7%)		34(13.5%)	14(12.4%)	20(14.4%)	
IIIA	36(14.2%)	17(13.7%)	19(14.7%)		37(14.7%)	16(14.2%)	21(15.1%)	
IIIB	54(21.3%)	27(21.8%)	27(20.9%)		57(22.6%)	28(24.8%)	29(20.9%)	
IVA	19(7.5%)	9(7.3%)	10(7.8%)		17(6.7%)	6(5.3%)	11(7.9%)	

NICT, neoadjuvant immunochemotherapy; BMI, body mass index; CR, complete response; PR, partial response; PD, progression disease; SD, stale disease; pCR, pathological complete response; LN, lymph node, *P-values were derived from Fisher’s exact test.

**Figure 2:**
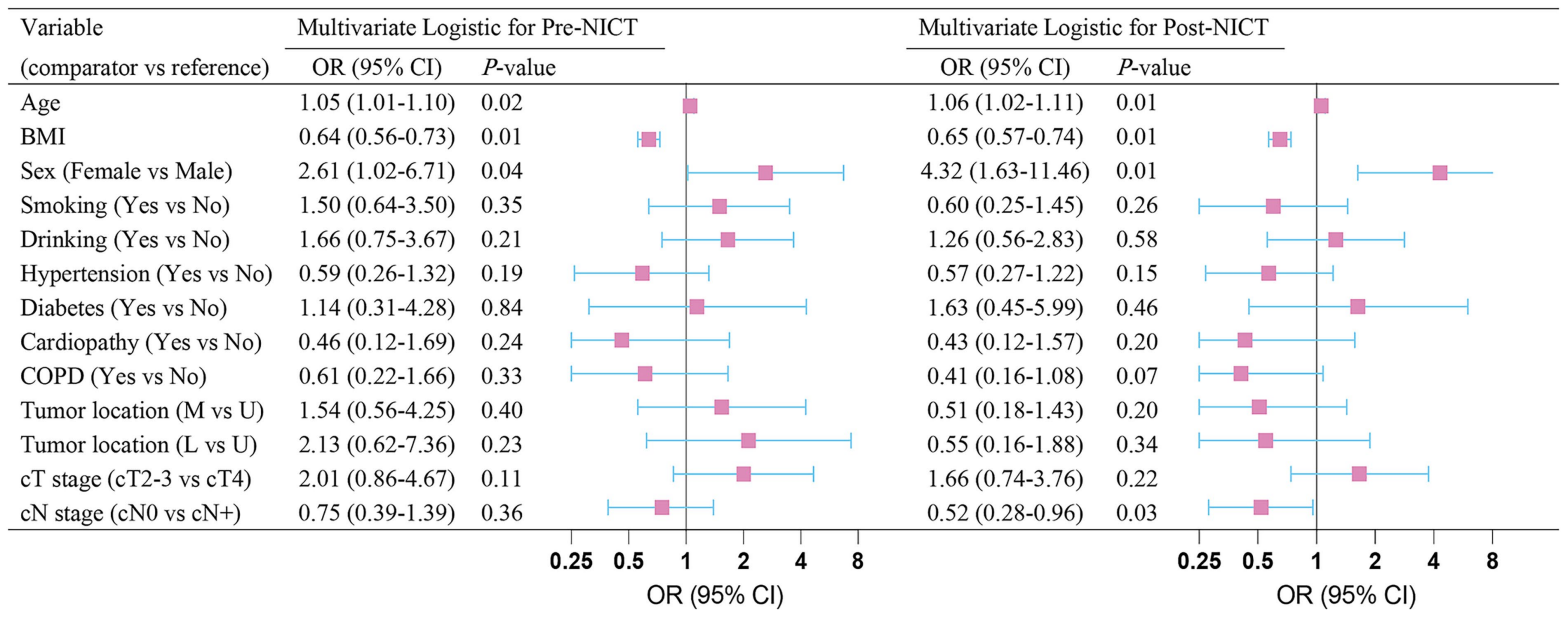
Multivariate logistic-regression analysis of sarcopenia before and after neoadjuvant immunochemotherapy.

In the multivariable binary logistic regression (forest plot in Figure 2), age (OR 1.05; 95% CI, 1.01–1.10; *p* = 0.02), BMI (OR 0.64; 95% CI, 0.56–0.73; *p* = 0.01), and male sex (OR 2.61; 95% CI, 1.02–6.71; *p* = 0.04), were identified as independent predictors of pre-NICT sarcopenia. Furthermore, age (OR 1.06; 95% CI, 1.02–1.11; *p* = 0.01), BMI (OR 0.65; 95% CI, 0.57–0.74; *p* = 0.01), male sex (OR 4.32; 95% CI, 1.63–11.46; *p* = 0.01), and clinical N0 stage (OR 0.52; 95% CI, 0.28–0.96; *p* = 0.03) emerged as independent predictors of post-NICT sarcopenia.

### Treatment-related adverse events and surgical complications

Patients with sarcopenia and those without demonstrated favorable safety profiles and manageable adverse events before and after NICT. As shown in Table 2, among grade 1–2 treatment-related adverse events before (145, 57.3%) and after (143, 56.7%) NICT, anemia was the most frequent, with a significantly higher incidence in the sarcopenia group than in the non-sarcopenia group (65.1% vs. 49.2%, *p* = 0.012; 63.3% vs. 48.7%, *p* = 0.024). The incidence of other TRAEs did not significantly differ between groups. In the pre- (30.8%) and post- (31.7%) NICT cohorts, postoperative pulmonary infection was the most common complication, with no significant difference between patients with sarcopenia and those without (*p* = 0.304; *p* = 0.972). We found no significant differences between groups in operative time, intraoperative blood loss, or anastomotic leakage (*p* > 0.05) (see Table 3).

**Table 2:** TRAEs of neoadjuvant therapy.

Variables	Total (*n* = 253)	Pre-NICT sarcopenia		*p*-value	Total (*n* = 252)	Post-NICT sarcopenia		*P*-value
		No (*n* = 124)	Yes (*n* = 129)			No (*n* = 113)	Yes (*n* = 139)	
Leukopenia								
None	190(75.1%)	98(79%)	92(71.3%)	0.137	187(74.2%)	90(79.6%)	97(69.8%)	0.075
Grade 1–2	58(22.9%)	25(20.2%)	33(25.6%)		59(23.4%)	21(18.6%)	38(27.3%)	
Grade 3–4	5(2.0%)	1(0.8%)	4(3.1%)		6(2.4%)	2(1.8%)	4(2.9%)	
Neutropenia								
None	219(86.6%)	111(89.5%)	108(83.7%)	0.175	216(85.7%)	102(90.3%)	114(82.0%)	0.059
Grade 1–2	23(9.1%)	9(7.3%)	14(10.9%)		24(9.5%)	8(7.1%)	16(11.5%)	
Grade 3–4	11(4.3%)	4(3.2%)	7(5.4%)		12(4.8%)	3(2.7%)	9(6.5%)	
Anemia								
None	106(41.9%)	62(50.0%)	44(34.1%)	0.012	107(42.5%)	57(50.4%)	50(36.0%)	0.024
Grade 1–2	145(57.3%)	61(49.2%)	84(65.1%)		143(56.7%)	55(48.7%)	88(63.3%)	
Grade 3–4	2(0.8%)	1(0.8%)	1(0.8%)		2(0.8%)	1(0.9%)	1(0.7%)	
Thrombocytopenia								
None	206(81.4%)	100(80.6%)	106(82.2%)	0.757	206(81.7%)	91(80.5%)	115(82.7%)	0.652
Grade 1–2	45(17.8%)	23(18.5%)	22(17.1%)		44(17.5%)	21(18.6%)	23(16.5%)	
Grade 3–4	2(0.8%)	1(0.8%)	1(0.8%)		2(0.8%)	1(0.9%)	1(0.7%)	
Liver Abnormalities								
None	195(77.1%)	93(75.0%)	102(79.1%)	0.660	199(79.0%)	85(75.2%)	114(82.0%)	0.191
Grade 1–2	55(21.7%)	29(23.4%)	26(20.2%)		51(20.2%)	27(23.9%)	24(17.3%)	
Grade 3–4	3(1.2%)	2(1.6%)	1(0.8%)		2(0.8%)	1(0.9%)	1(0.7%)	
Kidney Abnormalities								
None	239(94.5%)	114(91.9%)	125(96.9%)	0.103	240(95.2%)	105(92.9%)	135(97.1%)	0.144
Grade 1–2	14(5.5%)	10(8.1%)	4(3.1%)		12(4.8%)	8(7.1%)	4(2.9%)	
Grade 3–4	0	0	0		0	0	0	

TRAEs, treatment-related adverse events; NICT, neoadjuvant immunochemotherapy.

**Table 3:** Complications after surgical treatment.

Variables		Pre-NICT sarcopenia				Post-NICT sarcopenia			Total (*n* = 253)
		No (*n* = 124)	Yes (*n* = 129)	*p*-value	Total (*n* = 252)	No (*n* = 113)	Yes (*n* = 139)	*p*-value
Operation time, median, min, [IQR]	210(185–240)	205(185–237)	210(190–245)	0.255	209(185–240)	205(185–238)	213(190–244)	0.223
Blood loss, median, ml, [IQR]	100(60–100)	100(60–100)	100(62–100)	0.520	100(10–100)	100(55–100)	100(68–100)	0.640
Pulmonary infection								
No	175(69.2%)	82(66.1%)	93(72.1%)	0.304	172(68.3%)	77(68.1%)	95(68.3%)	0.972
Yes	78(30.8%)	42(33.9%)	36(27.9%)		80(31.7%)	36(31.9%)	44(31.7%)	
Anastomotic leakage								
No	245(96.8%)	120(96.8%)	125(96.9%)	1.000*	242(96.0%)	108(95.6%)	134(96.4%)	0.738
Yes	8(3.2%)	4(3.2%)	4(3.1%)		10(4.0%)	5(4.4%)	5(3.6%)	
Gastric emptying disorders								
No	247(97.6%)	120(96.8%)	127(98.4%)	0.439*	245(97.2%)	108(9.6%)	137(98.6%)	0.248*
Yes	6(2.4%)	4(3.2%)	2(1.6%)		7(2.8%)	5(4.4%)	2(1.4%)	
Respiratory failure								
No	251(99.2%)	122(98.4%)	129(100%)	0.239*	250(99.2%)	112(99.1%)	138(99.3%)	1.000*
Yes	2(0.8%)	2(1.6%)	0		2(0.8%)	1(0.9%)	1(0.7%)	

NICT, neoadjuvant immunochemotherapy, *P-values were derived from Fisher’s exact test.

### Survival outcomes and prognostic factors

**Figure 3:**
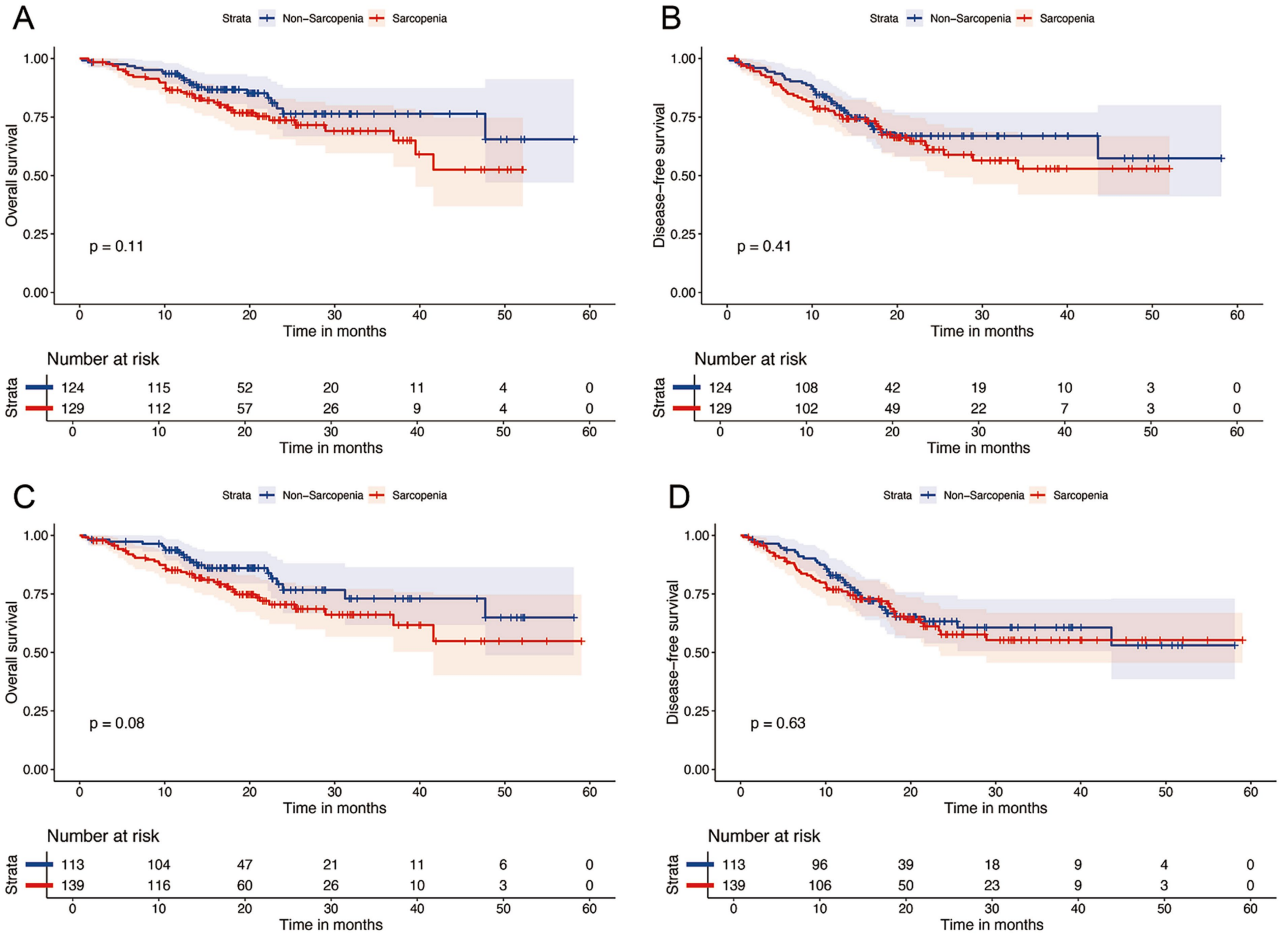
Kaplan–Meier survival analysis of OS **(A)** and DFS **(B)** between Pre-NICT sarcopenia and Pre-NICT non-sarcopenia; Kaplan–Meier survival analysis of OS **(C)** and DFS **(D)** between Post-NICT sarcopenia and Post-NICT non-sarcopenia.

**Figure 4:**
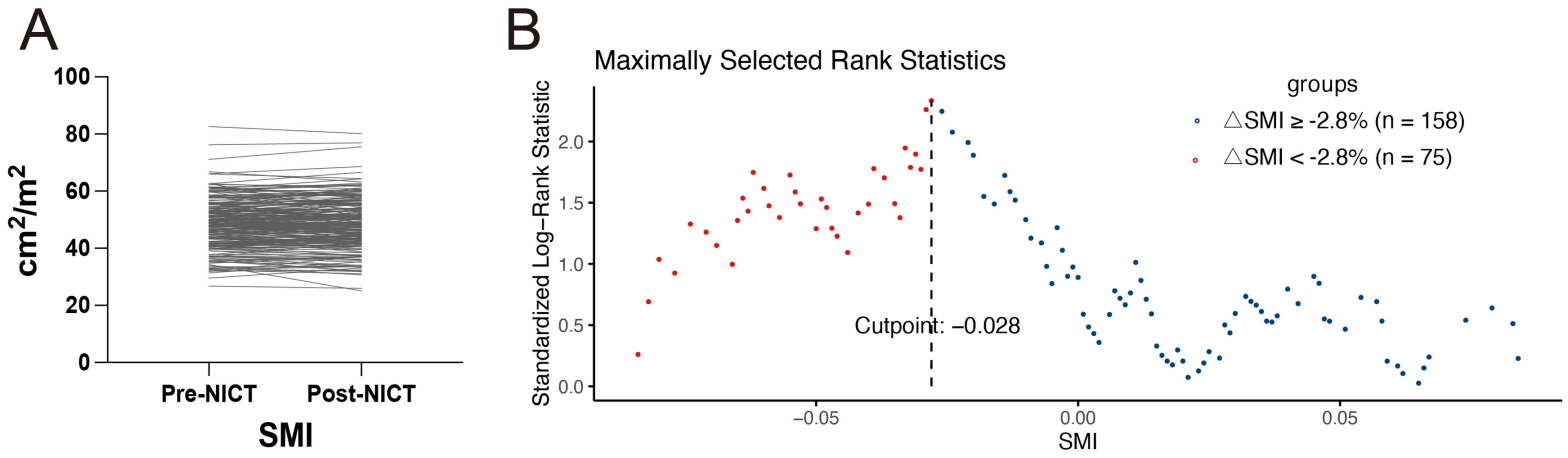
**(A)** Changes in skeletal muscle index of all patients before and after neoadjuvant immunochemotherapy. **(B)** The optimal cutoff value for OS determined using the Maxstat package in R software.

**Figure 5:**
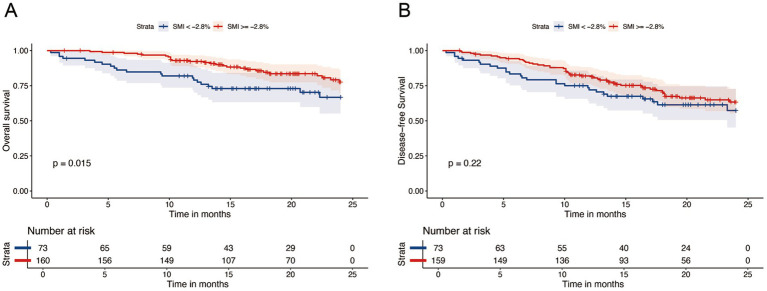
Kaplan–Meier survival analysis of OS **(A)** and DFS **(B)** between △SMI ≥ −2.8% and △SMI < −2.8%.

After a median follow-up of 20.4 months (95% CI: 19.2–21.6), 61 patients had died. The 1- and 2-year OS rates were 87 and 74%, respectively, whereas the DFS rates were 77 and 62%. Kaplan–Meier curve (Figure 3) shows that OS or DFS did not significantly differ between patients with sarcopenia and those without, either before or after NICT (*p* > 0.05). To assess the prognostic impact of dynamic skeletal muscle changes during NICT, we analyzed 233 patients with complete pre- and post-treatment imaging to calculate ΔSMI%. We used the Maxstat package in R to determine the optimal ΔSMI% cutoff for OS; a threshold of −2.8% (Figure 4) was used to stratify patients into low (<−2.8%) and high (≥−2.8%) ΔSMI% groups (see Supplementary Table 2 for baseline characteristics). Kaplan–Meier curve (Figure 5) shows that the 2-year OS rate was significantly higher in the ΔSMI% ≥ −2.8% group than in the ΔSMI% < −2.8% group (78% vs. 67%; *p* = 0.015). However, the 2-year DFS did not significantly differ between the two groups (63% vs. 57%; *p* = 0.22). Finally, we constructed a Cox proportional hazards regression model to further evaluate the prognostic value of relevant variables (Table 4). In the univariate analysis, age, chronic obstructive pulmonary disease, and ΔSMI% were associated with poorer OS; after multivariate adjustment, ΔSMI% remained an independent predictor of OS (high vs. low: HR 0.47; 95% CI, 0.25–0.91; *p* = 0.03).

**Table 4:** Univariate and Multivariate COX-regression analysis of risk factors for Overall Survival.

Variables	Univariate analysis		Multivariate analysis	
	HR (95% CI)	*p*-value	HR (95% CI)	*p*-value
Age	1.05 (1.00–1.10)	0.017	1.04 (1.00–1.09)	0.051
BMI	0.92 (0.83–1.02)	0.131		
Sex (reference: female)				
Male	1.32 (0.64–2.73)	0.456		
Smoking (reference: no)				
Yes	0.92 (0.52–1.64)	0.786		
Drinking (reference: no)				
Yes	1.02 (0.58–1.80)	0.95		
Hypertension (reference: no)				
Yes	1.43 (0.76–2.72)	0.27		
Diabetes (reference: no)				
Yes	1.57 (0.56–4.38)	0.389		
Cardiopathy (reference: no)				
Yes	2.03 (0.80–5.12)	0.136		
COPD (reference: no)				
Yes	2.13 (1.03–4.41)	0.042	2.02 (0.97–4.19)	0.06
Tumor location (reference: upper)				
Middle	0.98 (0.41–2.33)	0.962		
Lower	0.86 (0.29–2.56)	0.788		
cT stage (reference: cT4)				
cT2-3	0.67 (0.33–1.34)	0.256		
cN stage (reference: cN+)				
N0	0.63 (0.34–1.17)	0.143		
△SMI (reference: <−2.8%)				
≥−2.8%	0.50 (0.28–0.89)	0.015	0.54 (0.30–0.97)	0.04
Pre-NICT sarcopenia (reference: no)				
Yes	1.45 (0.81–2.62)	0.214		
Post-NICT sarcopenia (reference: no)				
Yes	1.45 (0.80–2.63)	0.222		

BMI, body mass index; cT, clinical tumor; cN, clinical node; SMI, skeletal muscle index; NICT, neoadjuvant immunochemotherapy.

### Comparison between the high-level (△SMI% > −2.8%) group and the low-level (△SMI% < −2.8%) group

Supplementary Table 2 shows that, apart from a significantly higher BMI in the high-level group versus the low-level group (23.2 [IQR 21.3–24.9] vs. 21.6 [19.8–24.5]; *p* = 0.01), baseline clinical characteristics were otherwise balanced between cohorts. Tumors in the high-level group were predominantly in the mid-esophagus (75.9%) and lower esophagus (15.8%), whereas those in the low-level group were mainly in the mid-esophagus (62.7%) and upper esophagus (21.3%). A significant difference in pCR rates was observed between groups (high vs. low: 22.8% vs. 12.0%; *p* = 0.05), with the high-level group exhibiting significantly better pathological downstaging after two NICT cycles (*p* = 0.03). In Supplementary Table 3, besides anemia (*p* = 0.01), leukopenia incidence also significantly differed between groups (*p* = 0.04). The high-level group had a lower rate of postoperative pulmonary infection than did the low-level group, although the difference was not statistically significant (28.5% vs. 37.5%; *p* = 0.17) (see Supplementary Table 4).

## Discussion

In this study, we investigated the impact of sarcopenia and skeletal muscle loss on short-term survival outcomes and treatment response in patients with ESCC during NICT. The results showed that excessive skeletal muscle loss during treatment was a negative prognostic factor, whereas sarcopenia diagnosed before or after neoadjuvant therapy was not. Furthermore, excessive muscle loss may help predict inferior treatment response and an increased incidence of hematologic toxicities.

In our cohort, sarcopenia prevalence increased from 50.9% pre-treatment to 55.1% post-treatment, consistent with prior studies (15, 17) and highlighting its high prevalence in esophageal cancer. Nonetheless, the prognostic relevance of sarcopenia in esophageal cancer remains debated (13, 14, 19, 20, 23). In a prior meta-analysis, sarcopenia was linked to worse DFS and OS outcomes (24). Our findings align with recent studies (19, 20, 23), showing that excessive muscle loss during therapy (not static sarcopenia status) was prognostically detrimental. Han et al. (23) found that excessive muscle loss during treatment was significantly associated with worse OS (HR 2.29; 95% CI 1.42–3.73; *p* = 0.001) and RFS (HR 1.62; 95% CI 1.12–2.35; *p* = 0.011). Additionally, Xiao et al. (25) reported a non-linear relationship between ΔSMI% during neoadjuvant chemoradiotherapy and survival outcomes (*p* < 0.05), with ΔSMI% ≥ 12% serving as an independent prognostic factor for OS and DFS (*p* = 0.04; *p* = 0.03). These discrepancies may result from variations in measurement techniques and diagnostic thresholds for sarcopenia, as well as patient selection across studies (26). In cases of ICI therapy, Ying et al. (19) reported that patients with positive ΔSMI% had better OS (*p* = 0.04). Thus, monitoring dynamic muscle loss may be more clinically relevant than that of static sarcopenia status in treatment planning. Our data show that a ΔSMI% threshold of −2.8% may be the most effective predictor of short-term OS under NICT.

The mechanisms by which sarcopenia influences ESCC progression and immunotherapy response remain unclear. A growing body of evidence indicates that skeletal muscle not only serves locomotor functions but also acts as an endocrine organ, regulating immune responses via paracrine secretion of myokines (27). In the context of immunotherapy, CD4^+^ and CD8^+^ T lymphocytes are the principal effector cells mediating antitumor activity (28). Notably, muscle-derived interleukin-15 (IL-15) has been shown to enhance CD8^+^T-cell survival and cytotoxicity (29). Conversely, when muscle mass is depleted, interleukin-6 (IL-6) levels become aberrantly elevated (30), which suppresses T-cell activation and proliferation, thereby weakening antitumor immunity. Furthermore, skeletal muscle loss is associated with decreased peripheral CD4^+^ T-cell counts (28, 31), suggesting a predisposition toward T-cell exhaustion. Together, these findings suggest that sarcopenia may undermine the effectiveness of immune checkpoint inhibitors by disrupting the IL-15/IL-6 balance and exacerbating T-cell dysfunction. In NSCLC immunotherapy settings, sarcopenia has been linked to hyperprogressive disease (32, 33). Moreover, Ying et al. (19) found significantly less muscle loss in the DC group compared with that in the non-DC group (*p* = 0.03) among patients with ESCC undergoing 3 months of NICT, consistent with our findings. We observed that before NICT, patients with sarcopenia had a significantly lower pCR rate than those without sarcopenia did, with a similar trend in the ΔSMI% < −2.8% subgroup. Based on these results, sarcopenia and its dynamic changes may serve as predictive biomarkers of ICI response.

Declines in skeletal muscle mass have been linked to higher rates of chemotherapy-induced cytotoxicity across various solid tumors (16, 34, 35). A prospective study in patients with NSCLC receiving first-line platinum chemotherapy showed that low muscle mass increased the risk of severe hematologic toxicity by approximately 2.5-fold (35). Furthermore, chemotherapy agents may exacerbate muscle atrophy, creating a vicious cycle (9). However, the literature remains controversial regarding whether sarcopenia increases treatment-related adverse events (36, 37). Our results reveal that excessive muscle loss during treatment significantly increases the risks of leukopenia (*p* = 0.04) and anemia (*p* = 0.01). Moreover, several studies (22, 33, 34) indicate that sarcopenia may be a risk factor for major postoperative complications. Zhang et al. (38) reported that, in older patients with ESCC, the sarcopenia group had significantly higher rates of postoperative pneumonia (29.8% vs. 16.9%; *p* < 0.01) and anastomotic leak (9.5% vs. 3.7%; *p* < 0.05) than did the non-sarcopenic group. In contrast to expectations, sarcopenia did not significantly predict postoperative complications in our study. Existing evidence on sarcopenia is primarily based on neoadjuvant chemoradiotherapy, and real-world data on the potential impact of ICIs on adverse events have not been recently reported. We observed a higher rate of pulmonary infections in the excessive muscle loss group, although the difference was not statistically significant (37.5% vs. 28.5%; *p* = 0.17).

This study has some limitations. First, although this clinical study is the largest to date assessing sarcopenia’s impact in ICIs-treated ESCC and the first in which ΔSMI% = −2.8% was proposed as a prognostic cutoff, our findings are based on single-center data and require multicenter prospective validation. Therefore, we defined ΔSMI% < −2.8% as excessive muscle loss to underscore the clinical relevance of treatment-induced sarcopenia. Second, with a median follow-up of 20.4 months, short-term outcomes were addressed; nonetheless, long-term efficacy remains unclear. The lack of detailed documentation of nutritional support (e.g., ONS utilization rates, achievement of caloric targets) may confound interpretations of muscle wasting; future investigations should prospectively standardize nutritional interventions to mitigate such confounding. Finally, no universally accepted method or standard exists for diagnosing sarcopenia. We adopted the common approach of calculating SMI from L3-level CT images, consistent with prior research.

## Conclusion

In summary, our findings show that in patients with locally advanced ESCC receiving ICIs, excessive skeletal muscle loss during treatment is associated with poorer short-term survival outcomes. Additionally, regarding therapeutic response and adverse effects, excessive skeletal muscle loss and sarcopenia could serve as predictive biomarkers for the efficacy and toxicity of ICIs. Furthermore, the pivotal role of multidisciplinary team (MDT) -including dietitians- in the perioperative multidisciplinary management of patients with ESCC, particularly in implementing standardized exercise interventions and nutritional support strategies, is highlighted in this study.

## Data Availability

The original contributions presented in the study are included in the article/Supplementary material, further inquiries can be directed to the corresponding authors.
