# Supplementary material for: Impact of skeletal muscle loss and sarcopenia on outcomes of neoadjuvant immunochemotherapy in esophageal squamous cell carcinoma
Source: Front Nutr. 2025 Sep 12;12:1650337. doi: 10.3389/fnut.2025.1650337 (PMC12463639; doi:10.3389/fnut.2025.1650337)
Supplement: Supplementary file 1 [file Table_1.DOCX]

Supplement Table.1 Clinical characteristics

| Variables | Total (n=272) | n (%) |
| --- | --- | --- |
| Age, mean ± SD, years | 65 ± 7.6 | - |
| BMI, median, [IQR] | 22.9 (20.8-24.9) | - |
| Sex |  |  |
| Male | 199 | 73.2% |
| Female | 73 | 26.8% |
| Smoking |  |  |
| No | 128 | 47.1% |
| Yes | 144 | 52.9% |
| Drinking |  |  |
| No | 145 | 53.3% |
| Yes | 127 | 46.7% |
| Hypertension |  |  |
| No | 216 | 79.4% |
| Yes | 56 | 20.6% |
| Diabetes |  |  |
| No | 254 | 93.4% |
| Yes | 18 | 6.6% |
| Cardiopathy |  |  |
| No | 258 | 94.9% |
| Yes | 14 | 5.1% |
| COPD |  |  |
| No | 243 | 89.3% |
| Yes | 29 | 10.7% |
| Tumor location |  |  |
| Upper | 33 | 12.1% |
| Middle | 194 | 71.3% |
| Lower | 45 | 16.5% |
| Clinical TNM stage |  |  |
| II | 100 | 36.8% |
| III | 121 | 44.5% |
| IVA | 51 | 18.8% |
| Clinical response |  |  |
| CR + PR | 170 | 62.5% |
| SD | 96 | 35.3% |
| PD | 6 | 2.2% |
| Pathological CR |  |  |
| pCR | 52 | 19.1% |
| Non-pCR | 220 | 80.9% |
| ypTNM stage |  |  |
| I | 117 | 43.0% |
| II | 39 | 14.3% |
| IIIA | 38 | 14.0% |
| IIIB | 58 | 21.3% |
| IVA | 20 | 7.4% |

BMI, body mass index; CR, complete response; PR, partial response；

PD, progression disease；SD, stale disease; pCR, pathological complete response

Supplement Table.2 Clinical characteristics

| Variables | △SMI | |  |
| --- | --- | --- | --- |
|  | △SMI < -2.8% (N =75) | △SMI ≥ -2.8% (N =158) | *P*-value |
| Age, mean ± SD, years | 66.3 ± 7.7 | 64.6 ± 7.4 | 0.10 |
| BMI, median, [IQR] | 21.6(19.8-24.5) | 23.2(21.3-24.9) | 0.01 |
| Sex |  |  |  |
| Male | 54(72.0%) | 123(77.8%) | 0.33 |
| Female | 21(28%) | 35(22.2%) |  |
| Smoking |  |  |  |
| No | 38(50.7%) | 68(43.0%) | 0.33 |
| Yes | 37(49.3%) | 90(57.0%) |  |
| Drinking |  |  |  |
| No | 39(52.0%) | 80(50.6%) | 0.89 |
| Yes | 36(48.0%) | 78(49.4%) |  |
| Hypertension |  |  |  |
| No | 58(77.3%) | 126(79.7%) | 0.73 |
| Yes | 17(22.7%) | 32(20.3%) |  |
| Diabetes |  |  |  |
| No | 68(90.7%) | 151(95.6%) | 0.15 |
| Yes | 7(9.3%) | 7(4.4%) |  |
| Cardiopathy |  |  |  |
| No | 72(96.0%) | 147(93.0%) | 0.56 |
| Yes | 3(4.0%) | 11(7.0%) |  |
| COPD |  |  |  |
| No | 67(89.3%) | 140(88.6%) | 1.00 |
| Yes | 8(10.7%) | 18(11.4%) |  |
| Tumor location |  |  |  |
| Upper | 16(21.3%) | 13(8.2%) | 0.02 |
| Middle | 47(62.7%) | 120(75.9%) |  |
| Lower | 12(16.0%) | 25(15.8%) |  |
| Clinical TNM stage |  |  |  |
| II | 26(34.7%) | 58(36.7%) | 0.55 |
| III | 32(42.7%) | 74(46.8%) |  |
| IVA | 17(22.7%) | 26(16.5%) |  |
| Clinical response |  |  |  |
| CR + PR | 46(61.3%) | 104(65.8%) | 0.52* |
| SD | 26(34.7%) | 51(32.3%) |  |
| PD | 3(4.0%) | 3(1.9%) |  |
| No.of LNs harvested, mean ± SD | 20.7 ± 10.8 | 20.7 ± 9.5 | 0.98 |
| Pathological CR |  |  |  |
| pCR | 9(12.0%) | 36(22.8%) | 0.05 |
| Non-pCR | 66(88.0%) | 122(77.2%) |  |
| ypTNM stage |  |  |  |
| I | 26(34.7%) | 72(45.6%) | 0.03 |
| II | 17(22.7%) | 14(8.9%) |  |
| IIIA | 14(18.7%) | 21(13.3%) |  |
| IIIB | 13(17.3%) | 40(25.3%) |  |
| IVA | 5(6.7%) | 11(7.0%) |  |

SMI, skeletal muscle index; BMI, body mass index; CR, complete response; PR, partial response；PD, progression disease；SD, stale disease; pCR, pathological complete response; LN, lymph node, *P values were derived from Fisher’s exact test.

Supplement Table.3 TRAEs of neoadjuvant therapy.

| Variables | △SMI | |  |
| --- | --- | --- | --- |
|  | △SMI < -2.8% (N =75) | △SMI ≥ -2.8% (N =158) | *P*-value |
| Leukopenia |  |  |  |
| None | 50(66.7%) | 125(79.1%) | 0.04 |
| Grade 1-2 | 23(30.7%) | 30(19.0%) |  |
| Grade 3-4 | 2(2.7%) | 3(1.9%) |  |
| Neutropenia |  |  |  |
| None | 62(82.7%) | 140(88.6%) | 0.16 |
| Grade 1-2 | 6(8.0%) | 15(9.5%) |  |
| Grade 3-4 | 7(9.3%) | 3(1.9%) |  |
| Anemia |  |  |  |
| None | 22(29.3%) | 78(49.4%) | 0.01 |
| Grade 1-2 | 52(69.3%) | 79(50.0%) |  |
| Grade 3-4 | 1(1.3%) | 1(0.6%) |  |
| Thrombocytopenia |  |  |  |
| None | 59(78.7%) | 132(83.5%) | 0.36 |
| Grade 1-2 | 15(20.0%) | 25(15.8%) |  |
| Grade 3-4 | 1(1.3%) | 1(0.6%) |  |
| Liver Abnormalities |  |  |  |
| None | 57(76.0%) | 125(79.1%) | 0.53 |
| Grade 1-2 | 16(21.3%) | 33(20.9%) |  |
| Grade 3-4 | 2(2.7%) | 0 |  |
| Kidney Abnormalities |  |  |  |
| None | 69(92.0%) | 152(96.2%) | 0.18 |
| Grade 1-2 | 6(8.0%) | 6(3.8%) |  |
| Grade 3-4 | 0 | 0 |  |

TRAEs, treatment-related adverse events; SMI, skeletal muscle index.

Supplement Table.4 Surgical and pathological outcomes

| Variables | △SMI | |  |
| --- | --- | --- | --- |
|  | △SMI < -2.8% (N =75) | △SMI ≥ -2.8% (N =158) | *P*-value |
| Operation time, mean ± SD, min | 224.6 ± 51.9 | 220.2 ± 48.3 | 0.53 |
| Blood loss, mean ± SD, ml | 108.3 ± 64.1 | 103 ± 58.8 | 0.57 |
| Pulmonary infection |  |  |  |
| No | 47(62.7%) | 113(71.5%) | 0.17 |
| Yes | 28(37.3%) | 45(28.5%) |  |
| Anastomotic leakage |  |  |  |
| No | 72(96.0%) | 153(96.8%) | 0.72* |
| Yes | 3(4.0%) | 5(3.2%) |  |
| Gastric emptying disorders |  |  |  |
| No | 72(96.0%) | 155(98.1%) | 0.39* |
| Yes | 3(4.0%) | 3(1.9%) |  |
| Respiratory failure |  |  |  |
| No | 74(98.7%) | 157(99.4%) | 0.54* |
| Yes | 1(1.3%) | 1(0.9%) |  |

SMI, skeletal muscle index, *P values were derived from Fisher’s exact test.

Supplement figure.1


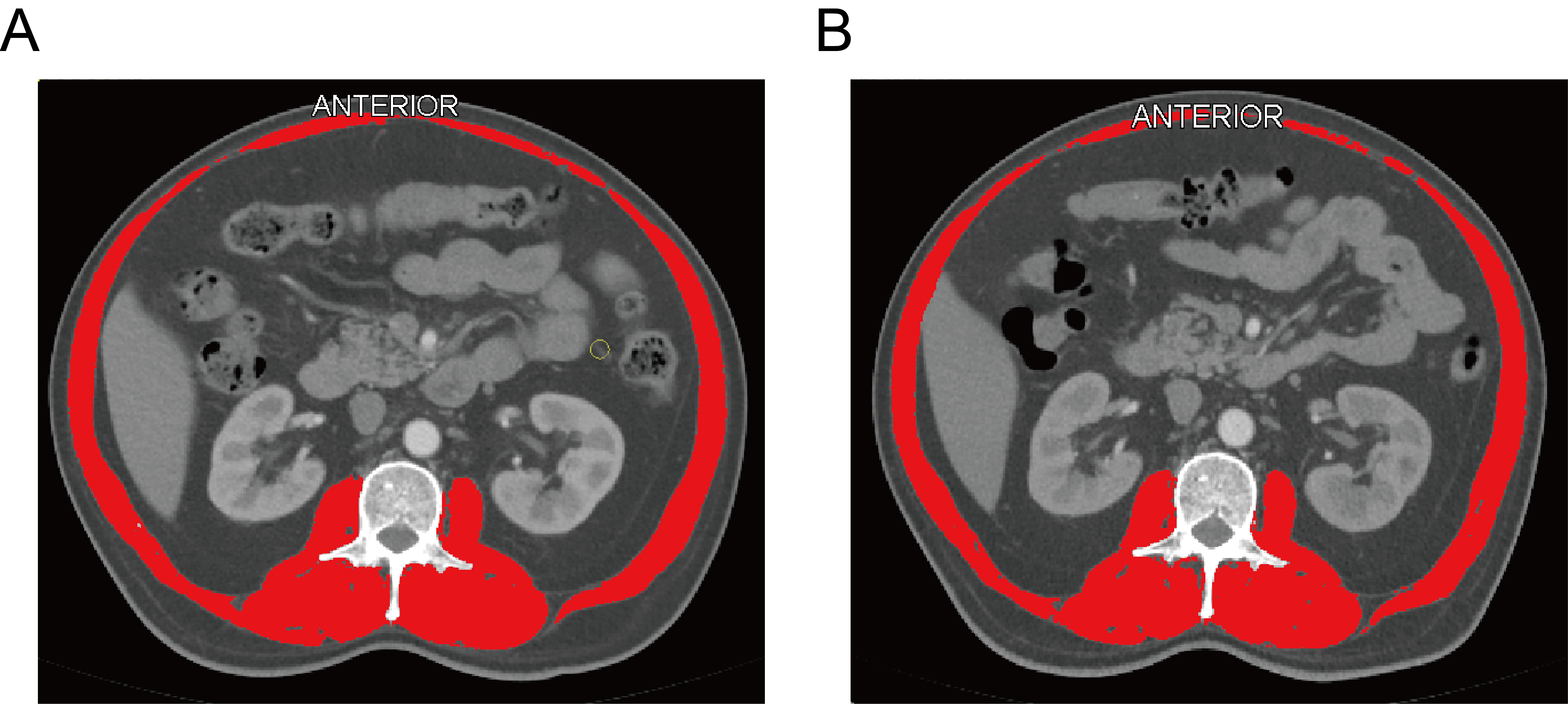


Supplement figure.1 The skeletal muscle contour area at the L3 level for the same patient before and after neoadjuvant immunochemotherapy. (A) Pre-NICT, SMI = 54.32 cm²/m²; (B) Post-NICT, SMI = 51.11 cm²/m².

Supplement figure. 2


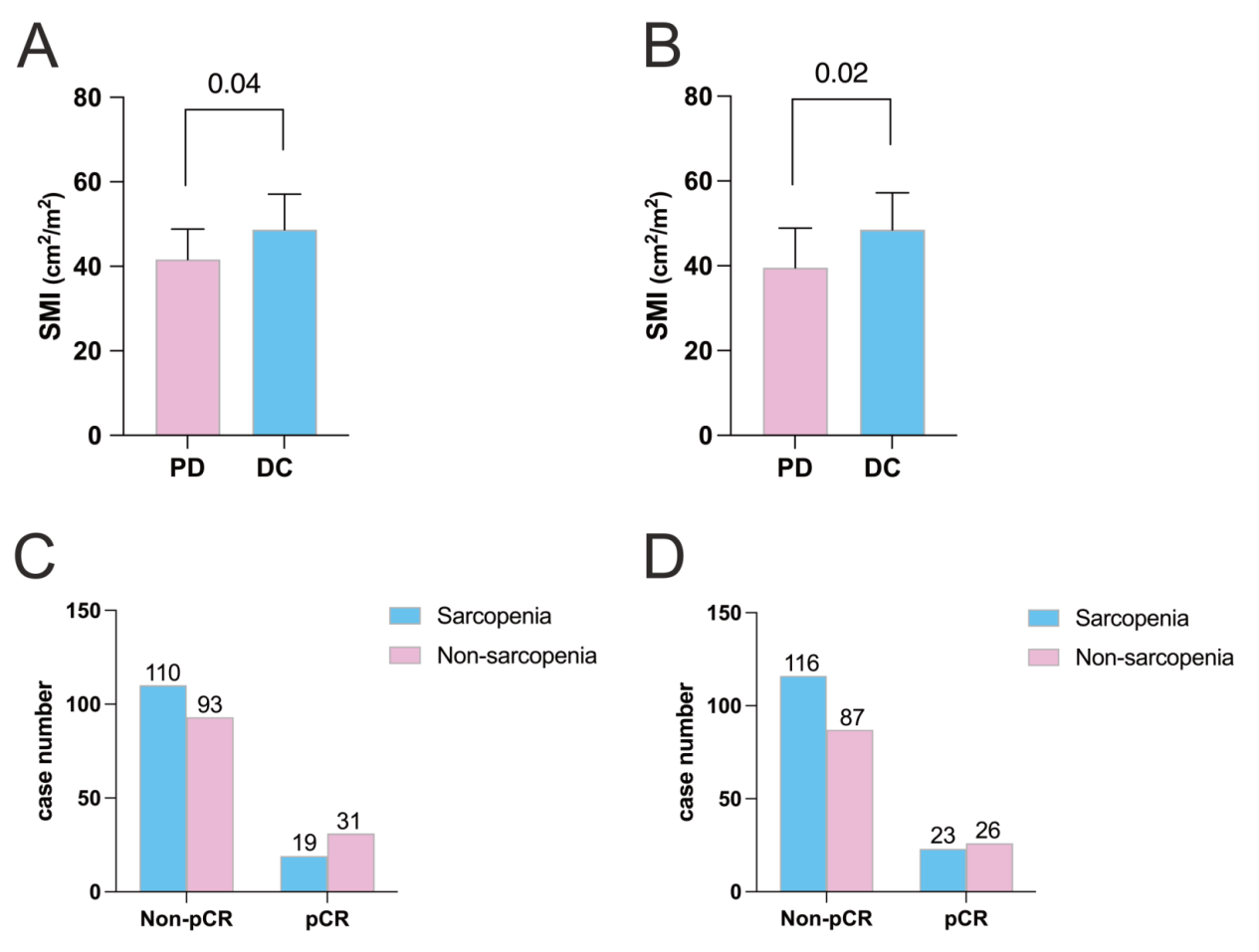


Supplement figure. 2 **(A)** Skeletal muscle area between the disease control (DC) and the progression disease (PD) before neoadjuvant immunochemotherapy; **(B)** Skeletal muscle area between the disease control (DC) and the progression disease (PD) after neoadjuvant immunochemotherapy; **(C)** Difference in pathological complete response (pCR) between sarcopenia and non-sarcopenia groups before neoadjuvant immunochemotherapy; **(D)** Difference in pathological complete response (pCR) between sarcopenia and non-sarcopenia groups after neoadjuvant immunochemotherapy.
